# Safety and efficacy of plasmapheresis in treatment of acute fatty liver of pregnancy—a systematic review and meta-analysis

**DOI:** 10.3389/fmed.2024.1433324

**Published:** 2024-10-18

**Authors:** Sujata Siwatch, Arka De, Bandhanjot Kaur, Divjot Singh Lamba, Simarpreet Kaur, Virendra Singh, Aravind Gandhi Periyasamy

**Affiliations:** ^1^Department of Obstetrics and Gynecology, PGIMER, Chandigarh, India; ^2^Department of Hepatology, PGIMER, Chandigarh, India; ^3^Department of Transfusion Medicine, PGIMER, Chandigarh, India; ^4^Department of Medicine, Microbiology and Infectious Diseases, University of Calgary, Calgary, AB, Canada; ^5^Department of Hepatology, PGIMER, Chandigarh, India; ^6^Department of Community Medicine, All India Institute of Medical Sciences, Nagpur, India

**Keywords:** acute fatty liver of pregnancy, plasmapheresis, plasma exchange, safety, efficacy, mortality, meta-analysis, biochemical improvement

## Abstract

**Introduction:**

Acute fatty liver of pregnancy (AFLP) is a fatal disease occurring in 3rd trimester. The safety and efficacy of plasmapheresis/plasma exchange (PP/PE) as an adjunctive treatment in patients of AFLP has been studied. We performed systematic review and meta-analysis to estimate the clinical parameters that included mortality rates and improvement of the biochemical parameters including Liver and Renal function enzymes, coagulopathy factors of AFLP patients.

**Methods:**

We searched PubMed, Ovid MEDLINE, Cochrane, CINAHL and Scopus, ClinicalTrials.gov. RevMan statistical software was used for meta-analysis.

**Results:**

Pooled survival proportion for AFLP patients treated with PP/PE was 87.74% (95% CI: 82.84 to 91.65). Efficacy of PP/PE was studied by its effect on mortality. PE/PP was associated with the reduction in the mortality with pooled odds ratio of 0.51 (95% CI: 0.08 to 3.09) with I^2^ = 86%. Sensitivity analysis after excluding outlier study, yielded a pooled odds ratio of 0.19 (95% CI: 0.02 to 1.52) with reduced heterogeneity (I^2^ = 63%). Biochemical parameter analysis demonstrated significant improvement post-PP/PE treatment, including decreased bilirubin (MD: 8.30, 95% CI: 6.75 to 9.84), AST (MD: 107.25, 95% CI: 52.45 to 162.06), ALT (MD: 111.08, 95% CI: 27.18 to 194.97), creatinine (MD: 1.66, 95% CI: 1.39 to 1.93), and Prothrombin time (MD: 5.08, 95% CI: 2.93 to 7.22).

**Discussion:**

Despite some heterogeneity, PP/PE shows promise in improving biochemical parameters in AFLP patients. PE can serve as a therapeutic approach for AFLP particularly in severe or refractory cases. PE provides the time for organ to recover and helps in creating a homeostatic environment for liver. Large RCTs and propensity matched studies are needed to better understand the safety and efficacy of the treatment.

**Systematic review registration:**

https://www.crd.york.ac.uk/prospero/display_record.php?ID=CRD42022315698.

## 1 Introduction

Acute fatty liver of Pregnancy (AFLP) is infrequent fatal hepatic dysfunction that is seen in early postpartum or 3^rd^ trimester of pregnancy. Its reported prevalence is 1/7000 to 1/20,000. It is associated with maternal mortality of 10–15% and perinatal mortality of 7–85% (1, 2). It was first described by Sheehan in 1940, as ‘acute yellow atrophy of the liver’ for AFLP (3). The prevalence in Indian population is not known (1). Though the exact cause of the AFLP is not yet clear, it may be related to the deficiency of the fatty acid oxidation enzymes in the fetus which may include SCAD (short chain acyl CoA dehydrogenase), LCHAD enzyme (Long chain hydroxy acyl-CoA dehydrogenase), MCAD (Medium Chain Acyl Dehydrogenase) and MTP (Mitochondrial trifunctional protein) which causes mitochondrial dysfunction leading to oxidative stress, in women developing of the AFLP.

AFLP is associated with clinical features like first pregnancy, male fetus, multiple pregnancies, preeclampsia, fetal fatty acid oxidation defects. AFLP often progresses to liver and renal failure, coagulopathy and metabolic dysfunction. The Swansea criteria are most widely used to diagnose AFLP (4). Timely termination of pregnancy and intensive medical support are necessary to ensure good maternal and foetal outcome. Intensive standard medical support usually includes transfusions for anaemia and coagulation deficiencies, hypoglycaemia and electrolyte correction and broad-spectrum antibiotic treatment. Recovery is slow especially with developing complications. For early diagnosis of AFLP, Goel et al. (5) proposed a ‘simple criterion’ to diagnose AFLP, i.e., Women in late pregnancy (Second or third trimester) with no explained cause of acute liver failure (i.e., jaundice in addition to coagulopathy and/or encephalopathy and/or hypoglycaemia).

Considering the high mortality, morbidity, liver and renal dysfunction, various supportive techniques have been used to offer interim support to allow liver recovery, to reduce hospital stay and thus improve the prognosis. These include Artificial liver support therapy (ALST) or Blood Purification Techniques. These modalities have shown significant efficacy as a bridge therapy, either aiding in spontaneous recovery or preparing patients for a liver transplant. ALST includes therapeutic plasma exchange (PE), Plasma Perfusion (PP), hemoperfusion and continuous renal replacement therapy (CRRT). Apheresis is the extracorporeal removal of blood constituents. Plasmapheresis or Plasma exchange is the apheresis technique in which plasma is removed from blood and remainder is returned to the body with the replacement fluid such as Albumin/Fresh Frozen Plasma conducted with clear therapeutic purpose by selectively eliminating or modifying the particular components present in the plasma. Replacement fluid is carefully chosen to address the underlying medical condition. Plasma perfusion is incorporating fresh frozen plasma in the body. CRRT is extracorporeal blood purification technique which aims to remove the excess fluid and blood solutes to treat Acute Kidney Injury.

Different modalities of CRRT are Slow continuous ultrafiltration, continuous veno-venous hemofiltration, continuous veno-venous haemodialysis, continuous veno-venous hemodiafiltration (6). In a systematic review by Tan et al. (7) use of PE in patients of acute liver failure improves survival and biochemical improvement. First performed by Russian physicians Vadim A Yurevich and Nikolay Konstantinovich Rosenberg (8) in 1913, Therapeutic plasma exchange (TPE) is carried out using two types of systems: membrane-based TPE (mTPE) and centrifugal-based TPE (cTPE). Membrane based TPE is based on molecular size and involves separating blood plasma from cellular components with the use of filter which removes the blood plasma and retains the cellular components. Heparin, an anticoagulant, is typically added to the blood before it is pumped through the filter. On the other hand, cTPE is based on molecular density and utilizes centrifugation to separate incoming whole blood into plasma, red and white blood cell components. Prior to centrifugation, citrate, an anticoagulant, is usually added. In both procedures, the remaining blood rich in cells is mixed with a replacement fluid (such as albumin or fresh frozen plasma) and returned to the patient to prevent hypovolemia (9). Membrane based TPE systems require calibration and thus more setup and priming time in contrast to cTPE (10–13). mTPE require high blood flow rate which may cause hemodynamic fluxes that could worsen the perfusion in the weakened hepatic microcirculation. mTPE is associated with higher incidence of clotting and cellular components loss due to limited pore diameter in the filter.

In this systematic review we aim to determine the safety and efficacy of PP/PE as an adjunctive treatment in acute fatty liver of pregnancy. Our objectives include effect of PE/PP on

1. Clinical parameters, i.e., mortality rates and length of the hospital stay of AFLP patients,
2. Improvement of the biochemical parameters including Liver function enzymes, renal function enzymes and coagulopathy factors.

## 2 Methods

### 2.1 Protocol registration

The protocol for this systematic review is registered with PROSPERO (International Prospective Register of Systematic Reviews; no. CRD42022315698.

### 2.2 Eligibility criteria

All the articles till September 2023 were included. As per the PICO, we included studies that reported patients of acute fatty liver of pregnancy (Population) who received plasmapheresis/PE as the treatment. Safety and efficacy of plasmapheresis was assessed by noting clinical outcomes like maternal mortality and hospital stay and biochemical parameters, i.e., The pre and post treatment change in biochemical profiles of the AFLP patients.

### 2.3 Information sources and literature search strategy

A systematic literature search was done from different databases such as PubMed, Ovid MEDLINE, Cochrane, CINAHL and Scopus, ClinicalTrials.gov. The search strategy included the terms (“plasmapheresis OR therapeutic plasma exchange/TPE, PLEX and acute fatty liver of pregnancy/AFLP”) and (Efficacy OR “effects”). Randomized control trials, observational studies, case series and case reports published in English language were included. Review articles and only abstracts were excluded during initial screening.

### 2.4 Study selection

Two reviewers independently screened the titles and abstracts of the retrieved records for eligibility (BK and SK). Any discrepancies were resolved by discussion or consultation with a third reviewer (SS). The full texts of potentially eligible records were obtained and assessed data extracted by BK and SK. Reasons for exclusion were recorded and reported.

### 2.5 Data extraction

Data extraction was done in structured manner to derive the following information: study author, year of publication, country of origin, study participants, clinical presentation (of subjects, age, gravida parity, gestational age, mode of delivery, foetal outcome, no. of days spent in hospital and ICU, biochemical profiling (haemoglobin, platelets, creatinine, AST, ALT, total bilirubin, PT) and plasma exchange procedure, indications for the plasma exchange, complications during the procedure etc. For comparison, the data was converted into same units. The studies were assessed for the quality and heterogeneity.

### 2.6 Quality assessment

Eligible studies included non-randomized controlled trials, prospective and retrospective cohort studies, case series, case reports that evaluated the use of plasmapheresis in the treatment of acute fatty liver of pregnancy. Each study’s quality was evaluated by quality assessment tools. ROBINS-1 tool (“Risk of bias in non-randomized controlled studies-of interventions”) was used for non-randomized controlled studies, i.e., case control studies. The tool assesses the studies in seven domains-confounding, selection, intervention classification, intervention deviation, missing data, outcome measurement and selective reporting. On the basis of these domains, studies are labelled as having low, moderate, serious, or critical risk of bias depending on the outcome studied (14). NIH quality assessment tool was used for observational studies (prospective or retrospective studies) (15). Quality assessment of the case reports was done by JBI critical appraisal tool (16). Authors assessed the risk of bias independently for each of the studies included. Disagreements were resolved among authors by a consensus.

### 2.7 Statistical analysis

Review Manager Software (RevMan 5.4,Cochrane Collaboration, Oxford, UK) and MedCalc statistical software was used for statistical analysis. Meta-analysis of the outcome variables was done to estimate the effect of PE/PP on maternal mortality. Odds Ratios and 95% CI were calculated. Random and fixed effects models were used to consider heterogeneity as applicable and the tests for heterogeneity of the studies was assessed using the I^2^ test. Sensitivity Analysis was conducted by removing the study with a high influence on the pooled estimate.

To study the pre and post intervention effect, Mean difference (MD) and 95% confidence interval (CI) was calculated. Random and fixed effects models were used to consider heterogeneity as applicable and the tests for heterogeneity of the studies was assessed using the I^2^ test (17). An I^2^ value of 0–39% was considered as non-significant heterogeneity; 40–75% as moderate heterogeneity; and 76–100% as considerable heterogeneity. A *p*-value > 0.05 was considered to reject the null hypothesis that the studies were heterogeneous. The studies with patient number (*n*) less than 2 were not included in the meta-analysis.

## 3 Results

### 3.1 Study selection

**Figure 1:**
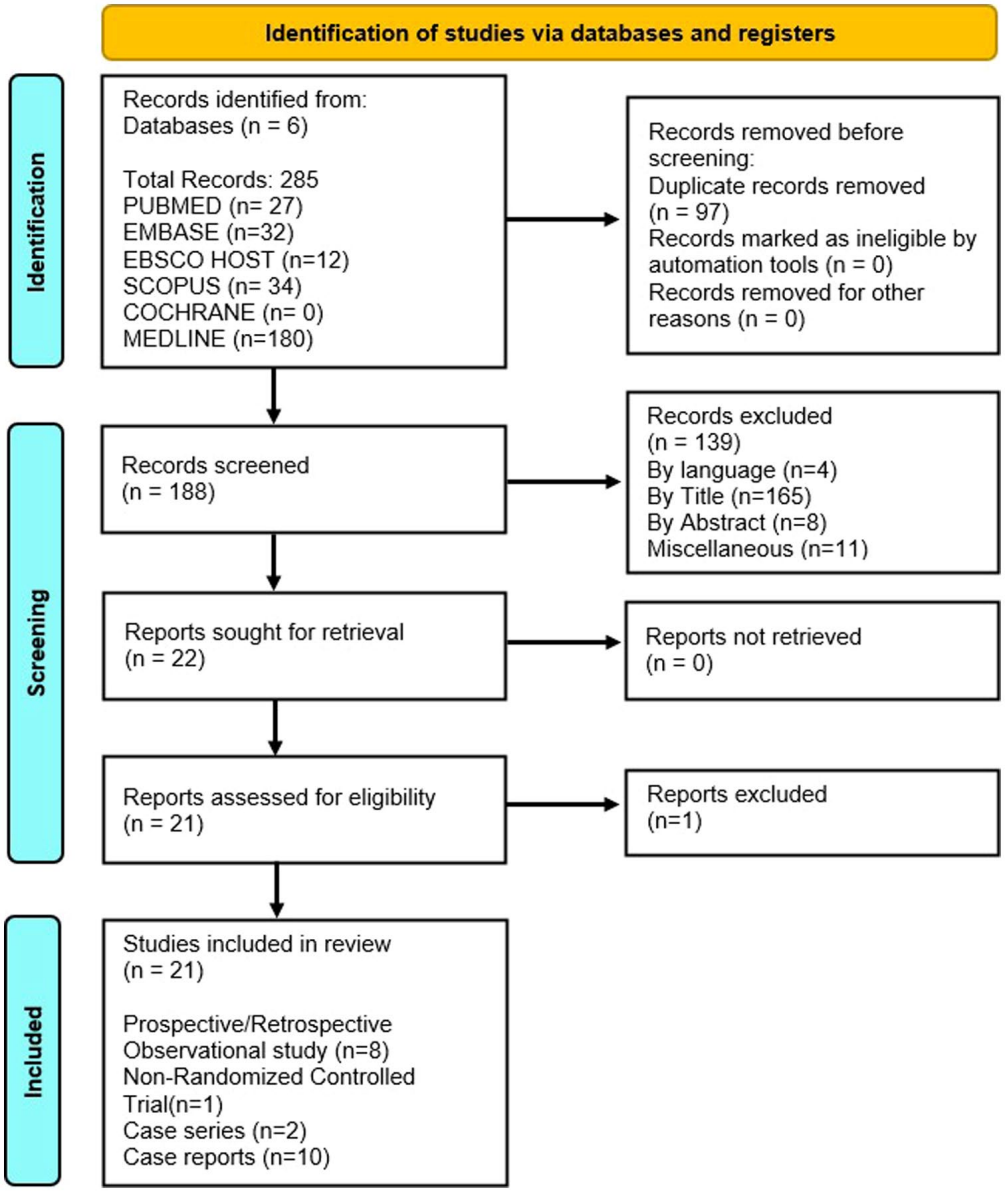
PRISMA Flow chart to show the selection process of the studies for the systematic review.

The literature search (detailed in Figure 1) resulted in identification of 285 published studies using the online database search of which 21 studies were included. Ten case reports, 2 case series, 8 observational studies and 1 non randomized control trial were assessed and finalized for eligibility (18–37). Sixteen were selected for the qualitative synthesis and 9 for quantitative synthesis (metanalysis; Figure 1). Searched articles were reported using the PRISMA checklist to ensure scientific precision. The PRISMA flow chart provides overview of the article selection process as shown in Figure 1. The meta-analysis contains 5 observational cohort studies and 1 case series. The studies included 10 studies from China (18, 19, 21–24, 33, 35–37), 2 studies from Japan (25, 26), 3 studies from USA (20, 34, 38), 1 study from North Africa (27), 2 studies from Iran (29, 32) and 1 study from India (31) and 2 from Morocco (28, 30) (Table 1).

**Table 1:** Characteristics of the included studies [author, year of publication,country of origin, total patients (*n*), age (years), mean gestational age (weeks), hospital and icu stay (days), maternal and fetal outcomes, maternal mortality (percentage and reason).

**Sr. no.**	**Author & country**	**Study design**	**Total** **patients** **(n)**	**Age (years)**	**Mean gestational age (n) & gravida and parity**	**Hospital** **stay (days)**	**ICU** **stay (days)**	**Fetal outcome & mode of delivery**	**Maternal mortality** **(n) and cause**
1	Li et al. (37), China	Retrospective Propensity Matched Cohort	298 Group A(PE)-79 Group B-(Non-PE)-211	27.21+/- 4.95	253.10+/-17.58 days; 1.69+/-0.81 Multiparae-57	NA	NA	Male-233 Multiplets-25; CS-267 Fetal death-78	50 In propensity matched 79 pairs PE group- 11 Control group- 22; Combined Encephalopathy and Postpartum Haemorrhage
2	Gao et al. (24), China	Retrospective Cohort	133 Group A (Non-PE+ CRRT)-92, Group B (PE and CRRT)-41	27+/-5.1	33.1; Primipara-95 Multipara-38	NA	NA	Male-75 Female-47 Intrauterine Death-9; CS-113 VD-20	22 Control group- 11 PE group-11
3	Jin et al. (36), China	Retrospective Cohort	39	26.4(22-31)	35.1(28-37); Primigravida-31 Multigravida-8	10-15	NA	Male-31 Female-14 CS-39	2
4	Tang et al. (18), China	Non RCT	Total- 28 Normal Control- 12	35 (28-40)	38 (36-40)	NA	NA	VD=1	NA
			AFLP PE- 13	33(26-41)	37 (32-40)	17 (12-26)	4 (2-9)	NA	NA
			AFLP case Control- 15	36 (28-39)	37 (33-39)	24 (18-34)	7 (3-19)	NA	NA
5	Ding et al. (21), China	Retrospective Case -Control	22 (Controls- 16,Cases-6)	27 (20-29)	36 (27-41); Primiparous-16 Multigravida-6	14	NA	Twins-2 VD-5, CS-14 Undelivered-3 Fetal death-3	14 (Control-13) (Case-1); Control (81.2%) 3-DIC; 3-MODS; 2-sepsis; 1-cerebral hemorrhage; 4-discontinued treatment and died due to complications Case-(16.7%) 1- MODS, DIC, Hepatic coma
6	Tang et al. (19), China	Prospective Observational Study	17	36(median)	37(32–40) (median); Primigravida-9 Multigravida-8	16 (9–27)	NA	Twins-2 Male-15 CS-16 VD-1	1 Secondary pulmonary infection Respiratory failure
7	Chu et al. (22), China	Retrospective Observational Cohort	11	26 (20-33)	33 (28-39); Primigravida-11	17 (9-38)	10 (4-23)	All LB CS-11	1 Septic Shock
8	Martin et al. (20), Mississippi USA	Retrospective Observational Cohort	6	23 (17-32)	36 (33-39); Primigravidas-5	21 (14-69)	No data	All LB CS-5 Vacuum birth by extraction-1	0
9	Yu et al. (23), China	Retrospective Observational Cohort	5	29 (23-36)	35 (29-39); Primiparous-2 Multiparous-3	25 (11-42)	9.4 (5-18)	All LB Male- 2 Female-3 CS-4 VD-1	0
10	Majidi et al. (32), Iran	Case series	3	Pt1-22 Pt2-32 Pt3-23	Pt1-37; G1P1 Pt2-34; G2P1 Pt3-36; G1P1	Pt1-20 Pt2-12 Pt3-8	Pt1-19 Pt2-12 Pt3-8	Pt1- Male Pt2- Female Pt3- Male CS-3	0
11	Wang et al. (35), China	Retrospective Case series	3	Pt1-29 Pt2-28 Pt3-32	Pt1-Primigravida, 38+3; Pt2-Primigravida,36+4; Pt3- G2P1,37+1;	All-10	NA	Male-3 CS-3	0
12	Kobayashi et al. (25), Japan	Case Report	1	21	33+4;G1P1	NA	60	Female-1 CS	0
13	Yamamoto et al. (26), Japan	Case report	1	35	33;G3P1	NA	54	LB, CS	0
14	Vives et al. (27), Costa Central america	Case report	1	33	40;Primigravida	NA	15	Female-1 CS	0
15	Aabdi et al. (28), Morocco	Case report	1	27	33;Primiparous	12	12	IUFD-1 VD	0
16	Ashrafganjoei et al. (29), Iran	Case report	1	26	34;G2P1L1	30	28	Male-1 CS	0
17	Rebahi et al. (30), Morocco	Case report	1	31	40;Primigravida	15	15	IUD-1 VD	0
18	Kumar et al. (31), India	Case report	1	22	36;Primigravida	21	NA	Male CS	0
19	Ye et al. (33), China	Case report	1	29	34.7;Multipara	35	NA	IUFD CS	0
20	Hartwell et al. (34), USA	Case report	1	19	26;Primigravida	13	NA	IUFD CS	0
21	Yang et al. (38), USA	Case report	1	41	31;G1P0	34	NA	Female CS	0

PE, Plasma Exchange; CS, Caesarean Section; VD, Vaginal Delivery; Pt, Patient; IUFD, Intrauterine Foetal Demise; LB, Live Born; FD, Foetal Demise; G, Gravida; P, parity.

### 3.2 Risk of Bias assessment

**Figure 2:**
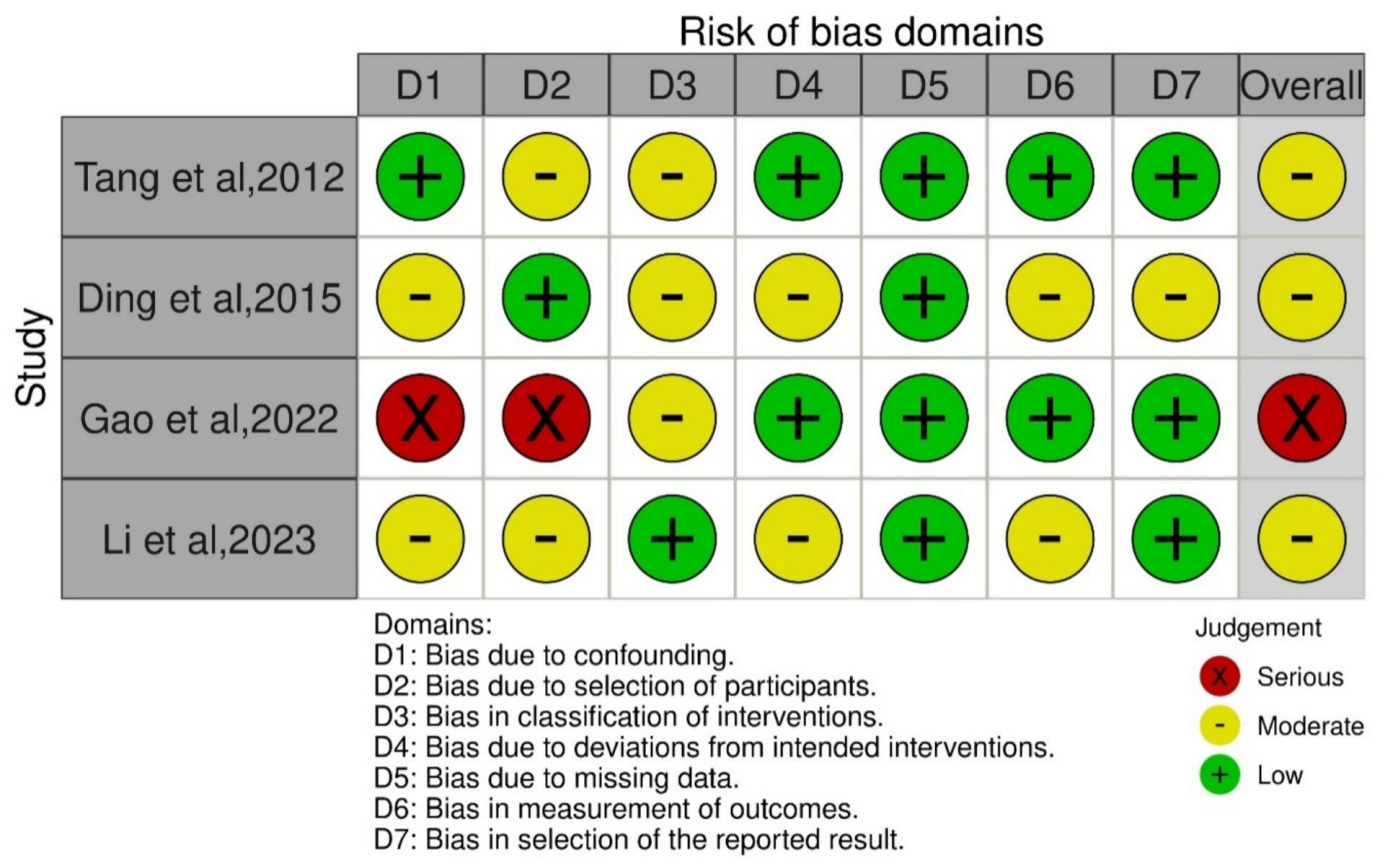
Graph of risk of bias assessment of the included case control studies using ROBINS-1 tool.

**Figure 3:**
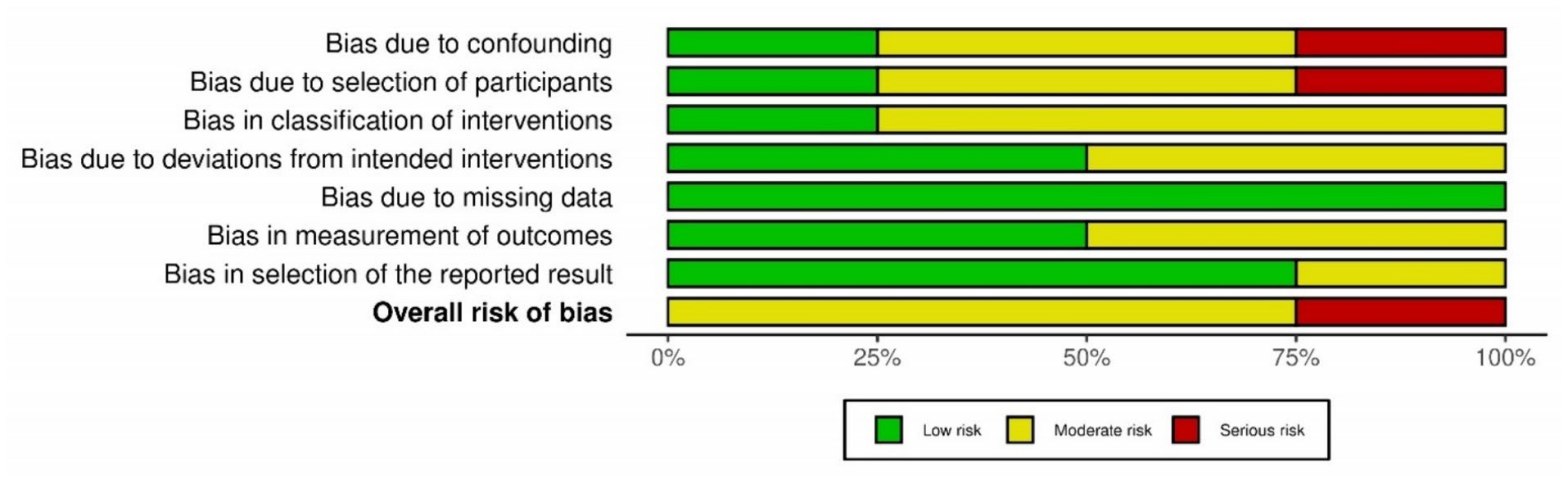
Summary of the risk of bias assessment of the included case control studies using ROBINS-1 tool.

The primary outcome of our study was safety of Plasmapheresis/Plasma exchange (PP/PE) with or without other liver support therapy in acute fatty liver of pregnancy patients. Quantitative data of mortality in the case control studies was collected and assessed using ROBINS-I tool (14). The risk of bias was variable among included studies. Three studies (18, 21, 37) were at moderate risk of bias and one study (24) was at serious risk of bias. Different biases of all the included non-randomized studies are depicted by the traffic light plot and weighted plot using the ROBINS (visualization tool) web application (39). They are shown in Figures 2, 3 respectively. The overall judgement on the risk of bias assessment for each domain in the included studies has been found to have moderate to serious risk. The secondary outcome was biochemical improvement with PE/PP. For the same, NIH quality assessment tool was used. Five observational studies (19, 20, 22, 23, 36) were assessed and all studies were of fair quality (Table 2). Quality Assessment of the case reports were from low to moderate (Supplementary Table 2).

**Table 2:** NIH quality assessment tool for observational cohort and cross sectional studies.

Sr. No.	Author	Year of publication	Q1	Q2	Q3	Q4	Q5	Q6	Q7	Q8	Q9	Q10	Q11	Q12	Q13	Q14	Quality rating
1	Chu et al. (22)	2012	Y	Y	Y	Y	CD	Y	Y	N	Y	N	Y	N	N	CD	Fair
2	Martin et al. (20)	2008	Y	Y	Y	Y	CD	Y	Y	N	Y	N	Y	N	N	CD	Fair
3	Yu et al. (23)	2014	Y	Y	Y	Y	CD	Y	Y	N	Y	N	Y	N	N	CD	Fair
4	Tang et al. (18)	2012	Y	Y	Y	Y	CD	Y	Y	Y	Y	Y	Y	N	N	CD	Fair
5	Jin et al. (36)	2012	Y	Y	Y	Y	CD	Y	Y	Y	N	Y	Y	N	N	CD	Fair

Quality was rated as 0 for poor (0–4 out of 14 questions), fair (5–10 out of 14 questions), good (11–14 out of 14 questions); NA, not applicable; NR, not reported. YOP, year of publication; Y, yes; N, no, NA, not applicable; CD, cannot determine; NI, no information. Q1: Was the research question or objective in this paper clearly stated? Q2: Was the study population clearly specified and defined? Q3: Was the participation rate of eligible persons at least 50%? Q4: Were all the subjects selected or recruited from the same or similar populations (including the same time period)? Were inclusion and exclusion criteria for being in the study prespecified and applied uniformly to all participants? Q5: Was a sample size justification, power description, or variance and effect estimates provided? Q6: For the analyses in this paper, were the exposure(s) of interest measured prior to the outcome(s) being measured? Q7: Was the timeframe sufficient so that one could reasonably expect to see an association between exposure and outcome if it existed? Q8: For exposures that can vary in amount or level, did the study examine different levels of the exposure as related to the outcome (e.g., categories of exposure, or exposure measured as continuous variable)? Q9: Were the exposure measures (independent variables) clearly defined, valid, reliable, and implemented consistently across all study participants? Q10: Was the exposure(s) assessed more than once over time? Q11: Were the outcome measures (dependent variables) clearly defined, valid, reliable, and implemented consistently across all study participants? Q12: Were the outcome assessors blinded to the exposure status of participants? Q13: Was loss to follow-up after baseline 20% or less? Q14: Were key potential confounding variables measured and adjusted statistically for their impact on the relationship between exposure(s) and outcome(s)?

### 3.3 Study characteristics

The key characteristics of the included studies are summarized in Tables 1, 3.

**Table 3:** Characteristics of the included studies [intervention group (*N*), blood purification treatment, type of technique & system used, indications for plex, procedures per patient, timing of pe, postpartum (days), complication during procedure, plasma volume exchanged; blood flow rate; plasma separation rate in each session and replacement fluid].

**Sr. no.**	**Author & YOP**	Intervention group (N)	**Blood purification treatment**	**Type Of technique & system Used**	**Conditions at/ Indications for blood purification treatment**	**Procedures per patient**	**Timing Of PE, post-partum (Days)**	**Complications during procedure**	**Plasma volume exchanged; blood flow rate; plasma separation rate in each session and replacement fluid**
1	Martin et al. (20)	6	PE	Centrifugal IBM/Cobe Model 2997 Cell Separator or more recently a Cobe Spectra Cell Separator	Worsening Coagulopathy (*n* = 6) Vomiting (*n* = 1) Increased Creatinine (*n* = 6) Hyperammonemia (*n* = 3) Hypoglycemia (*n* = 3) Blood pressure support with vasopressors (*n* = 2) Mental status change (*n* = 4) Increased blood product usage (*n* = 4) Fever (*n* = 2) Sepsis (*n* = 2) Ascites (*n* = 3) ARDS (*n* = 1) Mechanical Ventilation (*n* = 3)	2 to 4(mean-3)	4 (mean) range (2-9), mode day 3.	N=1 single episode of mild pulmonary edema	3-4 L; 50ml/kg; FFP.
2	Rebahi et al. (30)	13	PE	Membrane Continuous renal replacement therapy (CRRT) system (Diapact Multifunctional CRRT Machine, B. Braun, Melsungen, Germany). P2S membrane plasma separator (Fresenius, Bad Homburg,Germany	NA	1 to 3 Total-36 sessions	6 hours	Acute pulmonary edema (3/36) Hypocalcemia (10/36) Metabolic acidosis (6/36) Hypernatremia (4/36)	7200 ml (median) (range:3000-8600ml); 20 ml/min;80-100 ml/min FFP
3	Kumar et al. (31)	1	PE	NA	Continuous deterioration, Abdominal Distension, Worsening encephalopathy, Loss of deep tendon reflexes Worsening thrombocytopenia Cerebral edema,	5	4	Grade three ascites with pleural effusion	2L; 6 FFP+ 1 L isotonic 4% albumin solution
4	Majidi et al. (32)	1	PE	Centrifugal type apheresis system (Spectra Optia, Terumo BCT)	Persistent coagulopathy Thrombocytopenia Worsening renal function, Persistent organ dysfunction	NA	4	No periprocedural complications	2.6 L; 45ml/minute; FFP
5	Ashrafganjoei et al. (29)	3	PE	NA	Hepatic encephalopathy (*n* = 1) Severe respiratory distress (*n* = 1) Hepatic and renal failure(n=3) Hypoglycemia (*n* = 1) Diffuse edema (*n* = 1) Ascites (*n* = 2) Increased Bilirubin production (*n* = 1)	Pt1-22 Pt2-4 Pt3-3	Pt1-8 Pt2-6 Pt3-1	NA	Pt1- 3 L in 4 hours; 8 units FFP Pt2 – 3 L in 4 hours; 8 FFP + 100ml of 20% albumin PT3 - 3 L, FFP
6	Hartwell et al. (34)	1	PE	NA	Coagulopathy Severe edema, seizure	20	5	NA	NA
7	Córdoba-Vives et al. (27)	1	PE	NA	Altered mental status, Elevated ammonia Coagulopathy,	3	6	NA	FFP
8	Aabdi et al. (28)	1	PE	NA	Encephalopathy, Coagulopathy Persistent hypoglycemia	3	5	NA	NA
9	Jin et al. (36)	1	PE	NA	Neurological deterioration, Thrombocytopenia	5	2	NA	2L
10	Tang et al. (18)	37	PE	NA	Encephalopathy (*n* = 14) AKI (*n* = 19) DIC (*n* = 20)	1 to 4	1-5	NA	NA
11	Ding et al. (21)	6	PE with Plasma Perfusion	Membrane Based Plasmauto2IQ blood purification device (Diapact multifunctional CRRT; B. Braun, Melsungen, Germany) An OP208 membrane type plasma separator (Fresenius, Bad Homburg, Germany) Hemoperfusion cartridge as membrane filter	Not reported	Total-30 procedures, range(1 to 8)	14	No Complications	2.5-3.5 L For plasma perfusion-flow rate:180 ml/min with hemoperfusion cartridges at 40 ml/min; 5000 IU Heparin
12	Chu et al. (22)	11	PE Continuous hemodiafiltration	Membrane A multifiltrate machine (Fresenius Medical Care, Bad Homburg, Germany) was used for PE and CHDF. Plasm Flux PSu2S (Fresenius Medical Care) was used as the plasma-separating membrane. The polysulfone membrane hemofilter was used for CHDF (AV600S, 1.4 m2 surface area, Fresenius Medical Care).	Persistent coagulopathy, Increased creatinine (*n* = 11) ARDS (*n* = 4) Mental status change-(*n* = 4) Mechanical Ventilation required-(*n* = 4) Fever (*n* = 1) Sepsis (*n* = 1) Septic Shock (*n* = 1) Oliguria (*n* = 2) Blood pressure support with vasopressors (*n* = 2)	Total-45 sessions,2 to 8(range)	2 (Range-0-3)	PE+CHDF well tolerated. No Adverse effects reported.	3-4 L 80ml/min for PE 150 mL/min for CHDF 50ml plasma/kg for PE Dialyzer flow 20ml/kg/h and replacement fluid flow was set at 35 mL/kg/h for CHDF FFP for PE Bicarbonate-buffered hemofiltration fluid for CHDF
13	Yu et al. (23)	5	PE+ RRT	NA	Encephalopathy (*n* = 5) Haemorrhage (*n* = 1) Severe pneumonia (n=1) Fever (*n* = 2) Elevated creatinine(*n* = 5) Coagulopathy (*n* = 4) Oliguria (*n* = 2) Hepatorenal syndrome (*n* = 4) Sepsis(*n* = 1) ARDS (*n* = 1)	Total-13 session, 1 to 3 (range)	2	No complication/ Adverse side effects reported, Therapy well tolerated.	2.5-4 L 80-120 ml/ min 20ml/ min FFP and 20-40 g albumin
14	Tang et al. (19)	17	PE+CVVH	Continuous renal replacement therapy (CRRT) system (Diapact multifunctional CRRT, B. Braun, Melsungen, Germany For PE- P2S membrane plasma separator (Fresenius, Bad Homburg, Germany). For CVVH- AV 600S Polysulfone membrane (Fresenius)	Acute kidney Injury (n=17), DIC(n=7) Encephalopathy (n=9) Gastrointestinal hemorrhage (n=3) Acute pancreatitis (n=1)	2 to 3	6 hrs	For PE - Acute pulmonary Edema (n=7), Bleeding(n=3), Arrhythmia(n=1), Fever(n=2), Hypocalcemia (n=7), Metabolic Alkalosis (n=5), Hyperkalaemia(n=2), Hypernatremia (n=4). For CVVH- Bleeding(n=5), Hypotension(n=2), Arrhythmia(n=3), Hypocalcaemia (n=1), Metabolic Alkalosis (n=1)	5.5L to 8.6L (range) For PE- 80 ml/min For CVVH-200-250 ml/min 20ml/min FFP
15	Ye et al. (33)	1	PE+ renal replacement therapy	NA	Hypersomnia, decreased consciousness, increased serum creatinine and bilirubin Elevated serum lipase, amylase, respiratory failure, acute renal failure,	NA	7	No complications/Adverse side effects observed	FFP

PE, Plasma Exchange; CRRT, Continuous Renal Replacement Therapy; DIC, Disseminated intravascular Coagulopathy; ARDS, Acute Renal Distress Syndrome; RRT, Renal Replacement Therapy, FFP, Fresh Frozen Plasma; CHDF, Continuous hemodiafiltration; CVVH, Continuous venovenous Hemodiafiltration.

#### 3.3.1 Obstetrical information

In the 21 included studies, total cases of AFLP Patients were 575. The age range of the women was 19–41 years and the gestational age ranged from 26–40 weeks. Swansea score and simplified criteria were used to diagnose the AFLP patients.

#### 3.3.2 Foetal outcomes

Mode of delivery in most of studies was caesarean with notable more than 480 cases. From available data, 30 vaginal deliveries were reported in 6 studies (19, 21, 23, 24, 28, 30) while 94 cases of intrauterine fetal demise or fetal death were reported in 7 studies (21, 24, 28, 30, 33, 34, 37).

## 4 Results of the individual studies

### 4.1 Plasmapheresis/plasma exchange

16 studies detailed the information about the plasmapheresis/PE treatment. PE alone was given in 10 studies while PE with other Blood purification (BP) techniques like renal replacement therapy, hemofiltration, plasma perfusion was given in 6 studies. Six studies mentioned the techniques they used for the plasma exchange. 2 studies reported using centrifugal based plasma exchange system while 4 studies reported membrane-based plasma exchange system.

In the included studies, on average, plasmapheresis/PE was initiated within 4–8 days of the hospital admission. Procedures per patient varied depending upon the severity of the disease. Mostly, fresh frozen plasma was used as replacement fluid in PE (details in Table 3).

### 4.2 Indications for plasmapheresis/plasma exchange in AFLP patients

The most frequent reasons for initiating plasma exchange reported were abnormalities such as changes in sensorium and coma, persistent coagulopathy, advanced renal dysfunction and hepatic failure, fluid management issues such as significant ascites, oedema, anuria/oliguria, and/or fluid overload.

### 4.3 Meta-analysis results

#### 4.3.1 Maternal mortality

The main outcome of our study was to assess the safety and efficacy of PP/PE in terms of reducing mortality in AFLP patients. Survival with PE/PP as Adjunctive treatment were studied in 11 studies (18–24, 32, 35–37) with 223 AFLP patients. The studies with *n* < 2 were excluded in survival proportion analysis as they had no control arm. Metanalysis of the case reports and case series cannot be done as there are no comparable groups. There were 10 case reports and 1 case series. Five observational studies (19, 20, 22, 23, 36) had no control arm.

Pooled survival estimate was calculated. Pooled survival proportion for 223 patients with PE/PP as adjunctive treatment was 87.74% (95% CI: 82.84 to 91.65) under fixed effect model with I^2^ = 25.57% (95%CI: 0.00 to 63.11).

**Figure 4:**
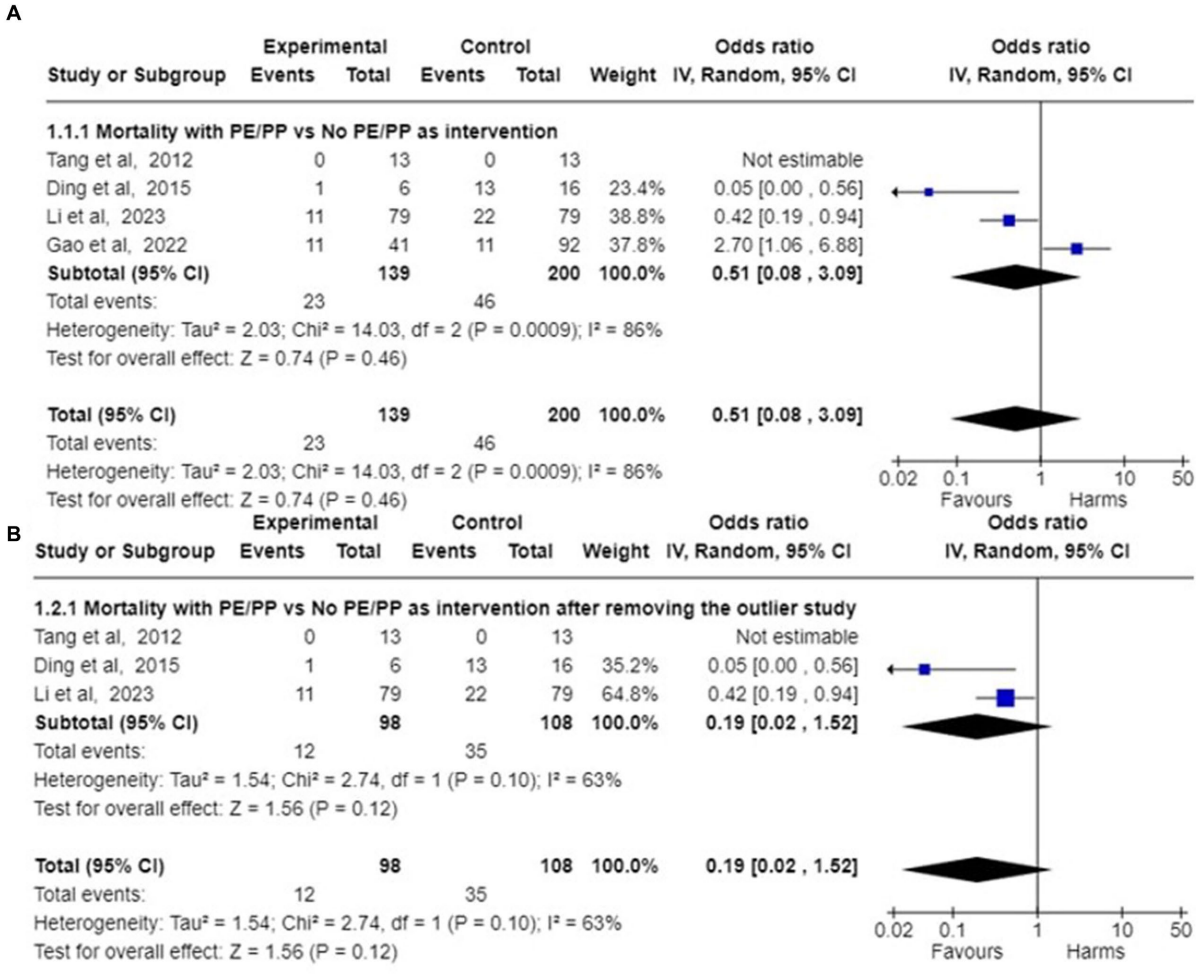
Forest plot of pooled odds ratio of mortality and their 95% confidence intervals (CI) and weights for individual studies. **(A)** Pooled Odds ratio of the mortality among AFLP patients given plasmapheresis/PE with or without other Blood Purification Technique as Intervention among 4 case control studies are depicted. **(B)** Pooled odds ratios of mortality among 3 case control studies are depicted after removing the outlier study (24).

A meta-analysis was conducted for four studies to assess the mortality on the use of Plasmapheresis/Plasma exchange (PP/PE) as adjunctive therapy on AFLP patients. Mortality data of the case control studies in the PE/PP group (Intervention) in comparison to control group is mentioned in Table 4. In the four included studies (18, 21, 24, 37), Pooled odds ratio and 95% confidence interval (CI) found that there is association between mortality and PP/PE as the adjuvant treatment. (Figure 2) PE/PP was associated with the reduction in the mortality with pooled odds ratio of 0.51(95% CI: 0.08 to 3.09) under random effect model. There is high heterogeneity among studies with Tau^2^ estimated as 2.03; Chi^2^ test estimated to be 14.03 with degree of freedom of 2 (*p* = 0.0009); I^2^ test estimated to be 86% as compared to no PP/PE as treatment (Figure 4).

**Table 4:** Mortality data of the case control studies in the PE/PP group (Intervention) vs. Control group.

Authors	**Year of publication**	Intervention		Controls		Risk of bias
		Mortality (*n*)	Total (*n*)	Mortality (*n*)	Total (*n*)	
Li et al. (37)	2023	11	79	22	79	Moderate
Ding et al. (21)	2015	1	6	13	16	Moderate
Gao et al. (24)	2020	11	41	11	92	Serious
Tang et al. (19)	2012	0	13	0	15	Moderate

#### 4.3.2 Cause of mortality

Causes of mortality are described Table 1. Tang et al. and Chu et al. reported the causes of mortality as septic shock and pulmonary failure, respectively. In study by Li et al., combined encephalopathy and post-partum haemorrhage were associated with maternal mortality (37). While Ding et al. observed multiorgan dysfunction and disseminated intravascular coagulation, sepsis, cerebral hemorrhage, hepatic coma associated with maternal mortality (21).

#### 4.3.3 Sensitivity analysis

A sensitivity analysis was performed to view any changes by omitting the outlier study. This was conducted by removing the outlier, i.e., Gao et al. (24) in which patients taken in PE group and non PE/PP were not comparable. In the study by Gao et al., patients treated with PE/PP had numerous poor prognostic factors, thus leading to higher maternal mortality as compared to the control group.

After removing the outlier study by Gao et al. (24), pooled survival proportion for 182 patients with PE/PP as adjunctive treatment was 90.37% (95% CI: 85.29 to 94.14) under fixed effect model with I^2^ = 0.00% (95%CI:0.00 to 42.09). Studies of Li et al., Ding et al. and Tang et al. (18, 21, 37) were analysed to assess the mortality in AFLP patients after the use of Plasmapheresis/Plasma exchange (PP/PE) as adjunctive therapy. The pooled odds ratio calculated came out to be 0.19 (95% CI: 0.02 to 1.52) under random effects model. There was still heterogeneity among studies with Tau^2^ estimated to be 1.54; Chi^2^ test estimated to be 2.74 with degree of freedom of 1 (*p* = 0.10); I^2^ test estimated the heterogeneity to be 63% (Figure 4).

### 4.4 Changes in biochemical parameters after plasmapheresis/plasma exchange

A meta-analysis was conducted to assess the effect of Plasmapheresis/Plasma exchange on acute fatty liver of pregnancy patients in terms of improvement in the biochemical profile. Table 5 mentions the quantitative values of the variables studied in the biochemical profile. Although included in the systematic review, the study by Tang et al. (19) was not included in the formal meta-analysis of change in biochemical parameters as data about standard deviation or error of relevant parameters were not reported in the study. Pooled standardized mean difference for different biochemical outcomes are as follows.

**Figure 5:**
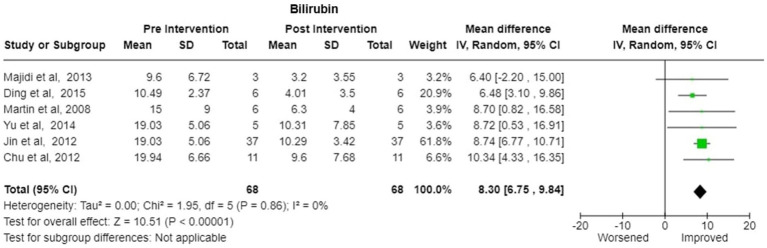
Bilirubin—forest plot showing mean difference (MD) and their 95% confidence interval (CI) and weights for individual studies from Pre and Post Intervention (plasmapheresis/PE with or without other blood purification techniques) obtained from six studies. MD values are depicted by green squares for each study with positive value indicating positive effect of the Intervention and diamond depicts pooled MD.

**Figure 6:**
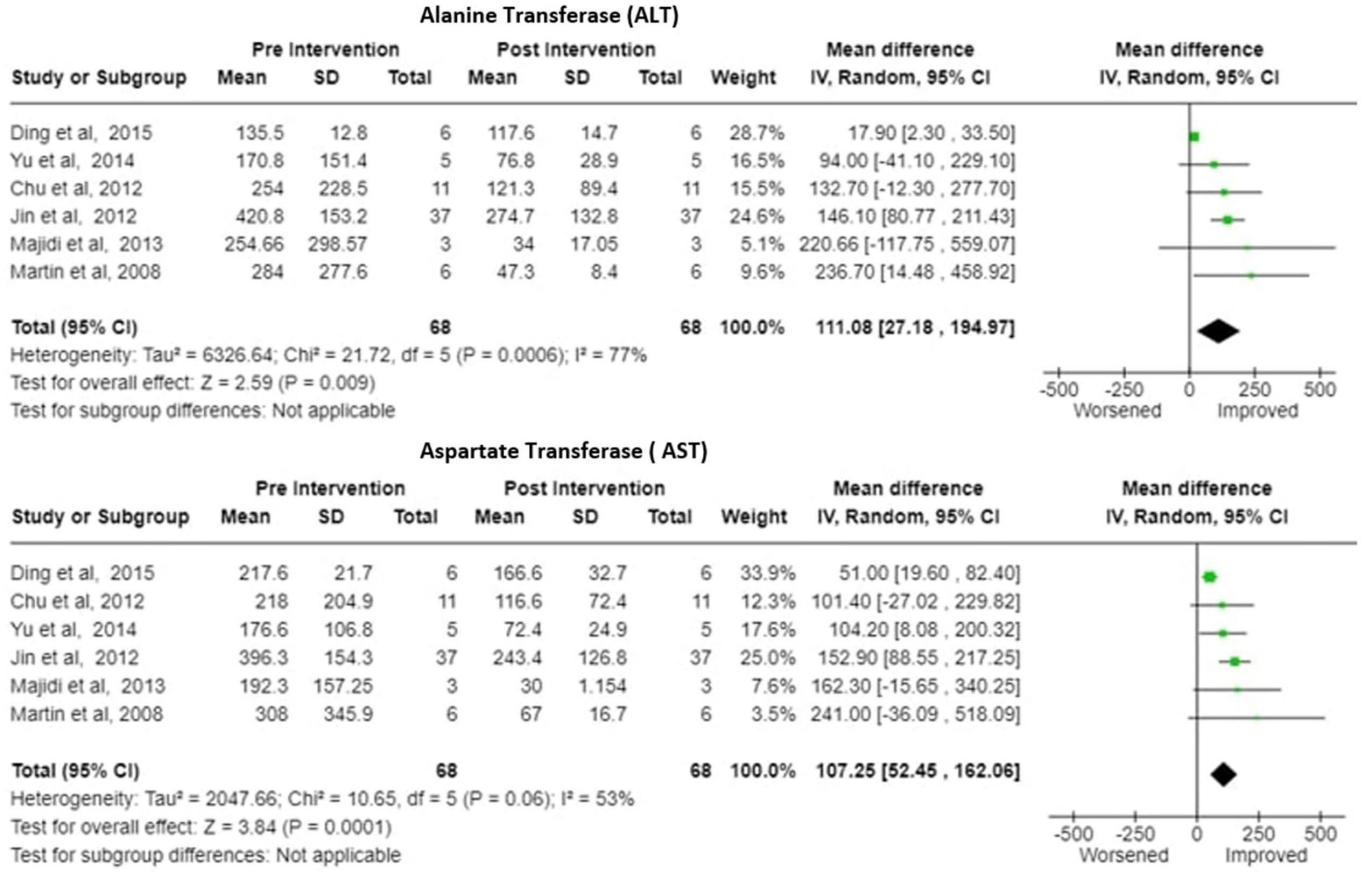
ALT (alanine transferase) and AST (aspartate transferase)—Forest plot showing pooled mean difference (MD) and their 95% confidence interval (CI) and weights for individual studies)from Pre and Post Intervention (plasmapheresis/PE with or without other blood purification techniques) obtained from six studies. MD values are depicted by green squares for each study with positive value indicating positive effect of the Intervention and diamond depicts pooled MD.

**Figure 7:**
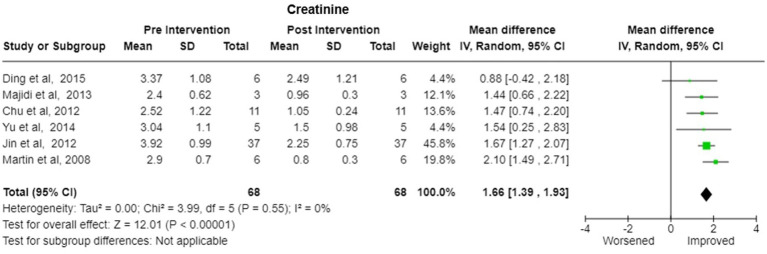
Creatinine—Forest plot showing pooled mean difference (MD) and their 95% confidence interval (CI) and weights for individual studies) from Pre and Post Intervention (plasmapheresis/PE with or without other blood purification techniques) obtained from six studies. MD values are depicted by green squares for each study with positive value indicating positive effect of the Intervention and diamond depicts pooled MD.

**Figure 8:**
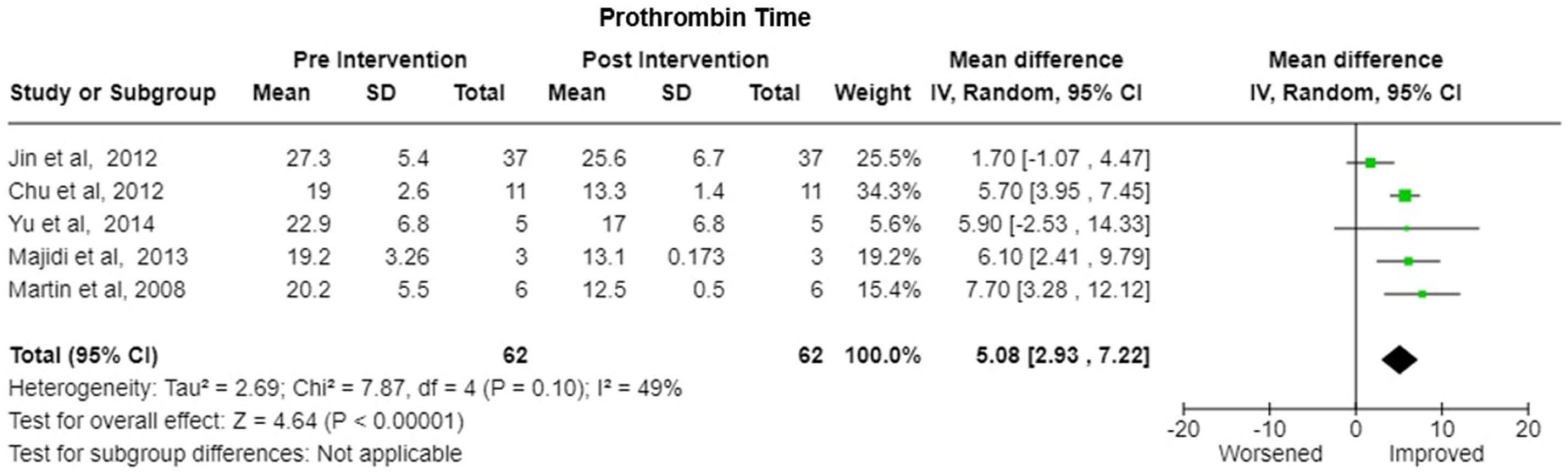
Prothrombin time—forest plot showing Pooled mean difference (MD) and their 95% confidence interval (CI) and weights for individual studies) from Pre and Post Intervention (plasmapheresis/PE with or without other blood purification techniques) obtained from six studies. MD values are depicted by green squares for each study with positive value indicating positive effect of the Intervention and diamond depicts pooled MD.

1. Bilirubin-Six studies reported the change in the bilirubin values after treatment. There was significant decrease in pooled bilirubin values after plasmapheresis treatment with mean difference (MD) of 8.30 (95% Cl: 6.75 to 9.84) using random effects model. There was no observed heterogeneity between studies with Tau^2^ estimated to be 0.00; Chi^2^ test estimated to be 1.95 with degree of freedom of 5 (*p* = 0.86) and I^2^ test estimated the heterogeneity to be 0% (Figure 5).
2. Alanine Transferase (ALT)- Six studies reported the change in the ALT levels after the treatment A decrease in pooled ALT values with MD of 111.08 (95% CI:27.18 to 194.97) under random effects model. There was heterogeneity among studies with Tau^2^ estimated to be 6326.64; Chi^2^ test estimated to be 21.72 with degree of freedom of 5 (*p* = 0.0006 and I^2^ test estimated the heterogeneity to be 77% (Figure 6).
3. Aspartate Transferase (AST)- Analysis of six studies reporting the change in the AST levels after the treatment demonstrated a substantial decrease in pooled AST values after plasmapheresis treatment with MD of 107.25 (95% CI:52.45 to 162.06) under fixed effects model. There was heterogeneity among studies with Tau^2^ estimated to be 2047.66; Chi^2^ test estimated to be 10.65 with degree of freedom of 5 (p = 0.06 and I^2^ test estimated the heterogeneity to be 53% (Figure 6).
4. Creatinine-Pooled Creatinine values also showed improvement with the plasmapheresis with MD of 1.66 (95% CI: 1.39 to 1.93) under random effect model. There was heterogeneity among studies with Tau^2^ estimated to be 0.00; Chi^2^ test estimated to be 3.99 with degree of freedom of 5 (*p* = 0.55) and I^2^ test estimated the heterogeneity to be 0% (Figure 7).
5. Prothrombin Time (PT)- Five studies reported the change in the prothrombin time levels after the treatment. Pooled Prothrombin Time showed some improvement after the plasmapheresis with MD of 5.08 (95% CI: 2.93 to 7.22) under random effects model. There was heterogeneity among studies with Tau^2^ estimated to be 2.69; Chi^2^ test estimated to be 7.87 with degree of freedom of 4 (*p* = 0.10) and I^2^ test estimated the heterogeneity to be 49% (Figure 8).

**Table 5:** Comparison of clinical parameters pre-plasma exchange vs. post-plasma exchange data with/without another blood purification technique as intervention in acute fatty liver of pregnancy (AFLP) patients during hospital stay.

**Sr.** **no.**	**Author**	**Total** **Patients** **(n)**	**Blood** **Purification** **Technique**	**Total** **Bilirubin** **(mg/dl)**		**AST** **(IU/L)**		**ALT** **(IU/L)**		**Creatinine** **(mg/dl)**		**PT (sec)**		**Platelets** **(10** ^**3**^ **/mm** ^**3**^ **)**		**Hb** **(g/dl)**	
				Pre	Post	Pre	Post	Pre	Post	Pre	Post	Pre	Post	Pre	Post	Pre	Post
1	Majidi et al.	3	PE	3.6	1	370	32	596	53	1.9	1.3	18	13	60	120	9.2	9.9
				16.9	7.3	136	30	126	20	2.2	0.9	16.7	13	63	190	9.5	9.5
				8.5	1.3	71	30	42	29	3.1	0.7	22.9	13.3	39	220	6	12
2	Kumar et al.	1	PE	11.8	8.5	77	52	60	32	1.6	0.7	-	-	45	52	6.7	8.8
3	Martin et al.	6	PE	15	6.3	308	67	284	47.3	2.9	0.8	20.2	12.5	46.3	195	-	-
4	Rebahi et al.	1	PE	20.6	16.75	325	98	190	33	34	7	20	9.5	75	256	12.5	7.8
5	Aabdi et al.	1	PE	69	-	300	70	325	69	41	13	-	-	57	72	10	9.0
6	Jin et al.	39	PE	19.03	10.29	396.3	243.4	420.8	274.7	3.92	2.25	27.3	25.6	61.6	63.2		
7	Chu et al.	11	PE + CHDF	19.94	9.6	218	116.6	254	121.3	2.52	1.05	-	-	-	-	-	-
8	Ding et al.	6	PE+ PP	10.49	4.01	217.6	166.6	135.5	117.6	3.37	2.49	-	-	-	-	-	-
9	Yu et al.	5	PE + RRT	19.03	10.31	176.6	72.4	170.8	76.8	3.04	1.50	-	-	-	-	-	-
10	Tang et al.	17	PE+CVVH	14.03 (median)	2.69 (median)	261 (median)	80 (median)	146 (median)	66 (median)	2.59 (median)	2.05 (median)	21.1 (median)	11.6 (median)	88 (median)	160 (median)	-	-

AST, aspartate transferase; ALT, alanine transferase; PT, prothrombin time; Hb, hemoglobin; PE, plasma exchange; CHDF, continuous hemodiafiltration; RRT, renal replacement therapy; PP, plasma perfusion.

## 5 Complications during the plasma exchange/plasmapheresis

PE/PP appears to be generally well tolerated when used to treat with ALF and ACLF patients. The side effects of PE/PP may include sepsis, port-related infection, vein inflammation, bleeding, accidental arterial puncture, hypotensive and hypothermia and side effects by citrate anti-coagulation include hypocalecemia, muscle pain, arrythmia etc. Transfusion-related acute lung injury (TRALI) and acute pulmonary odema may arise due to FFP administered as a replacement during PP/PE. But the incidence of these pulmonary complications has decreased overtime due to female plasma being discarded as a risk reduction strategy or diverted for plasma fractionation (40, 41).

In this review, complications were studied/reported in four studies (18–20, 30). These included mild pulmonary oedema and grade 3 ascites with pleural effusion, hypocalcaemia, metabolic acidosis and hypernatremia which can be due to FFP.10 studies did not report complications or adverse side effects during procedures (21–23, 27–29, 31–34).

Tang et al. (19) compared various complications between PE and CVVH, respectively, (continuous venovenous hemofiltration) and reported a incidence of acute pulmonary oedema (7/17 vs. 0/17, *p* value of 0.007 and hypocalcaemia (7/17 vs. 1/17, *p* value-0.039) respectively. Other complications like bleeding, arrhythmia, metabolic acidosis, fever, hyperkalaemia, hypernatremia, hypotension was also reported but not statistically significant.

## 6 Discussion

The present systematic review demonstrates the potential role of plasma exchange in the treatment of acute fatty liver of pregnancy after delivery.

The main outcome of our study was safety and efficacy of the plasma exchange in reducing mortality of the patients. With advancement in the diagnosis and management of AFLP, the maternal mortality rate is now estimated to be 12.5 to 18% (42, 43).

Pooled survival proportion of patients (*n* = 223) from included studies was 87.741% and after removing the outlier study of Gao et al. (24), we observed an impressive pooled survival rate of >90% for AFLP patients (*n* = 182), despite the poor liver function status. Our metanalysis of pooled odds ratio suggests that there is reduction in the mortality with PP/PE treatment. But wide confidence interval and high heterogeneity among studies suggests variability in effect sizes across the studies. Even after removing the outlier study, there is still some degree of heterogeneity. So the findings should be interpreted cautiously. PE/PP has been widely used in the management of liver failure of other causes and can also improve the outcome of the AFLP patients.

Another outcome was change in the biochemical parameters with PP/PE as adjunctive treatment. In our meta-analysis, six studies were incorporated. Pooled mean difference (MD) between biochemical outcomes (Bilirubin and creatinine) showed significant decrease with no heterogeneity among studies. Pooled MD of other biochemical outcomes (AST, ALT, Prothrombin Time) also showed improvement but with some heterogeneity among studies. Plasma exchange (PE) can be beneficial for patients with severe illnesses as it promotes the faster normalization of liver and renal enzymes. However, for patients with less severe conditions, supportive therapy alone may be sufficient.

Prompt induction of labor and termination of the pregnancy is the definitive and vital in the obstetrical management. Though normal vaginal delivery is generally considered safer, Caesarean section usually reduces the time to deliver as compared to induction of labour and vaginal delivery. In their systematic review, Wang et al. studied the association between caesarean section and vaginal delivery and reported that maternal mortality was 44% lower (RR,0.56 [CI-0.41-0.76] in caesarean section although not statistically significant (44). AFLP is often complicated by hepatic encephalopathy, coagulopathy, multiorgan dysfunction syndrome, renal dysfunction, DIC, hypoglycaemia, septic shock, haemorrhage.

Guidelines by CSOG MFM Committee of China for clinical management of acute fatty liver of pregnancy includes the use of artificial liver treatment for patients with severe AFLP (45). Recently published guidelines by the European Association for the Study of Liver (EASL) and Japan Society of Blood Purification in Critical Care (JSBPCC), Indian National Association For Study Of The Liver (INASL) recommend using extracorporeal blood purification devices, including PE, for acute fulminant liver failure. However, the American Association for the Study of Liver Diseases (AASLD) has been more cautious and has not found enough solid evidence to routinely recommend the use of external artificial (sorbent-based) or bioartificial liver support systems (cell-based) in the management of acute liver failure (ALF) (46).

Tang’s study elaborates the effect of the PE on the molecular and cellular level and how PE can help lessen the hepatic injury in AFLP. Increased fatty acids in AFLP leads to excessive intake by hepatocytes, that stimulates the expression of reactive oxygen species, mitochondrial DNA mutations, and apoptosis. Dysfunction of the synthesis and detoxification function of liver leads to more accumulation of the toxic metabolites. PE significantly enhanced mitochondrial functionality by regulating mitochondrial membrane potential (MMP) and inhibiting oxidative stress responses and caspase-9 activation, resulting in a reduction in apoptosis in AFLP patients, the effect increasing, following several sessions (18).

It should be noted that most of the included cohort studies are retrospective in nature. Jin et al. treated 39 AFLP patients with PE and it was noted that the patient’s general condition improved after the first PE session (36). Additionally, Kumar et al. observed that PE led to significant improvements in renal and liver biomarkers (31).

Safety of PE also needs to be studied. PE is associated with complications during treatment like hypocalcaemia and metabolic acidosis, ascites, hypernatremia, bleeding, arrhythmia, fever, hyperkalaemia and complications like acute pulmonary oedema/TRALI due to FFP transfusion during PE/PP. But the incidence of these pulmonary complications has decreased overtime due to female plasma being discarded as a risk reduction strategy or diverted for plasma fractionation (40, 41). Tang et al. (19) study observes that the use of PE alone can induce pulmonary oedema, secondary to the substantial requirement of the fresh frozen plasma. A major concern associated with PE is the administration of large doses of citrate anticoagulants, which can lead to hypernatremia, hypocalcaemia, and metabolic alkalosis. Hypocalcaemia can be prevented during PE by prophylactic calcium administration and calcium monitoring (47). When treated with low-volume PE, citrate toxicity is generally seen less due to the low volumes of plasma exchanged (thus using less citrate) and the low rates of flow of the processed blood (using PE/PP centrifugal technique) in liver failure patients (48). In several reports, hemodynamic instability was seen as a contraindication to using PE/PP (49, 50). PE/PP in hemodynamically unstable patients raises concerns that it could exacerbate and have negative impact on patient outcomes. Active sepsis is regarded as a PE contraindication. It’s probable that PE’s suppression of the immune system’s overreaction will make sepsis worse (48). Optimal situation for starting PE may be in the golden window of sterile inflammation (51). PLEX use to treat liver failure is contraindicated if there has recently been a gastrointestinal bleeding (50). In the RCT by Larsen et al. for ALF patients (*n* = 92) there was no statistical difference between the complications who received high volume plasma exchange vs. standard medical treatment. PE/PP can be considered safe and tolerable for acute liver failure patients. Choice of the treatment depends on the severity of the condition (52). There is not much evidence to opine on whether normal volume or high volume exchange benefits more in AFLP patients. Plasmapheresis combined with other blood purification techniques have also been attempted in studies. Ding et al. observed that kidney and liver biochemical functions significantly enhanced when PE and Plasma perfusion were given for 2 weeks. The combination of plasma exchange (PE) and plasma perfusion (PP) in patients with liver disease enables the efficient removal of a significant quantity of toxic substances, as well as the improvement of clotting factors and albumin levels (21). Yu et al. used PE with renal replacement therapy such as continuous venovenous hemofiltration, continuous venovenous hemodiafiltration or continuous venovenous haemodialysis once on every other day. For severe AFLP patients with potentially fatal illness, plasma exchange and renal replacement therapy can be used for treatment of patients that do not respond to that conventional therapy (23). In the study by Tang et al. combining PE and continuous venovenous hemofiltration (CVVH), PE clears the bilirubin significantly than CVVH (*p* = 0.000). Plasmapheresis (PE) effectively eliminates circulating endotoxins and facilitates the replacement of coagulation factors and proteins, thereby correcting hepatic encephalopathy (HE). CVVH improves the renal functions by clearance of creatinine, inflammatory mediators, nitrogenous metabolic waste etc. (19). Yamamoto et al. and Ye et al. also endorse the same (26, 33).

Furthermore, it is worth noting that initiating plasmapheresis earlier appears to enhance its effectiveness and reduce the number of required sessions. Overall, these studies collectively suggest that plasma exchange, particularly when initiated promptly, can be effective in improving the clinical outcomes of severely ill AFLP patients, leading to favourable changes in various biochemical markers.

## 7 Limitations

Our study is limited by the quality of studies and heterogeneity in reporting. Published data on the patient outcomes with AFLP are mostly case reports, with no RCTs. Larger prospective studies may elucidate the impact of plasma exchange on the maternal survival. Clinical heterogeneity was a notable challenge. There were limited available studies on the topic and variations in study designs. MELD score for degree of liver failure is also not mentioned. The lack of standardized PE protocols, data collection methods and the absence of consistent reporting of treatment outcomes, use of different statistical measures made it challenging to ascertain the precise differences in treatment effects. While some case studies did provide pre-and post-plasma exchange data, it is crucial to consider that combining data from case reports may introduce biases and limitations, compromising the overall validity of the systematic review. The majority of studies lacked control groups. In order to address these limitations, the systematic review had to carefully consider the available evidence, focusing on the similarities and differences in study design, patient characteristics, and reported outcomes, while acknowledging the inherent limitations and potential biases associated with the included studies.

## 8 Conclusion

To conclude, emerging evidence suggests that PE can serve as a therapeutic approach for acute fatty liver of pregnancy (AFLP), particularly in severe or refractory cases. PE provides the organ with an opportunity to recover by ameliorating liver injury and creating a homeostatic environment conducive to hepatocyte regeneration. Better designed and larger randomized controlled trials or at the very least propensity matched retrospective or prospective cohort studies are the need of the hour, to gain granular understanding about the efficacy and patient selection for plasmapheresis/plasma exchange in acute fatty liver of pregnancy patients.

## Data Availability

The raw data supporting the conclusions of this article will be made available by the authors, without undue reservation.
