# Supplementary material for: Safety and efficacy of plasmapheresis in treatment of acute fatty liver of pregnancy—a systematic review and meta-analysis
Source: Front Med (Lausanne). 2024 Oct 18;11:1433324. doi: 10.3389/fmed.2024.1433324 (PMC11527697; doi:10.3389/fmed.2024.1433324)
Supplement: Supplementary file 2 [file Data_Sheet_2.docx]

**SUPPLEMENTARY FIGURES**


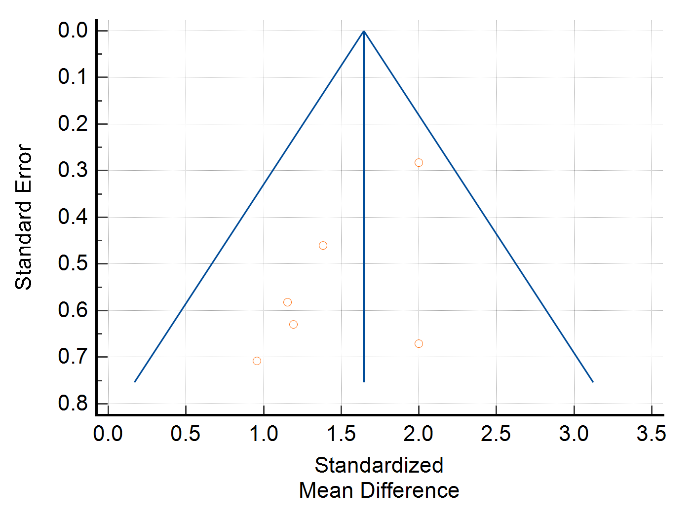


Supplementary Figure 1 - Funnel Plot of the studies showing publication bias by Egger’s test for the outcome Bilirubin after the treatment with PP/PE.


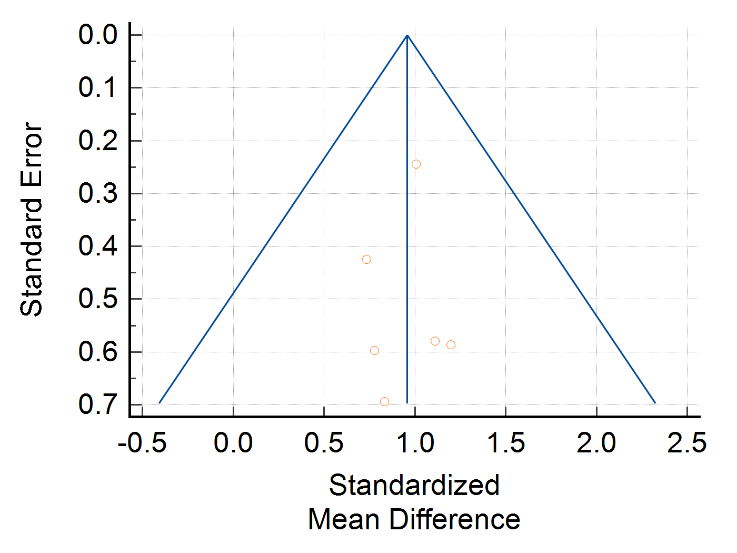


Supplementary Figure 2 - Funnel Plot of the studies showing publication bias by Egger’s test for the outcome Aspartate transferase after the treatment with PP/PE.


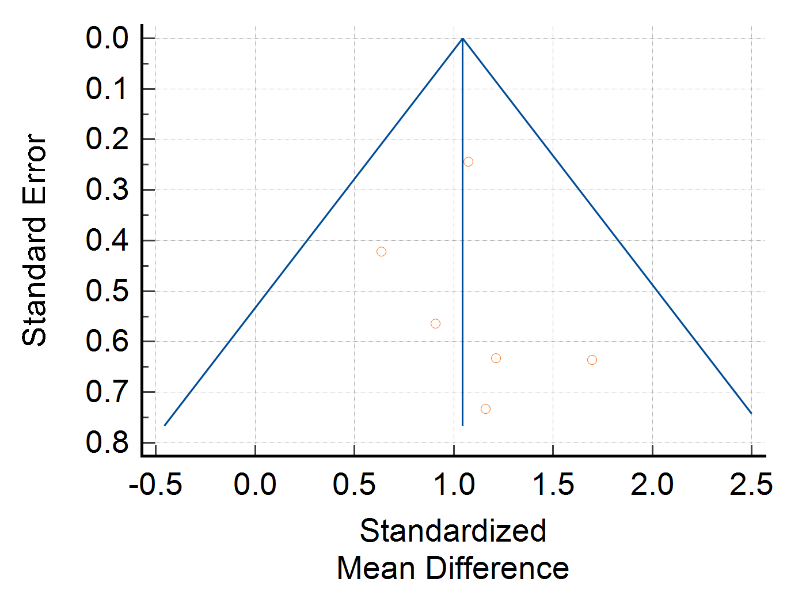


Supplementary Figure 3- Funnel Plot of the studies showing publication bias by Egger’s test for the outcome Alanine Transferese after the treatment with PP/PE.


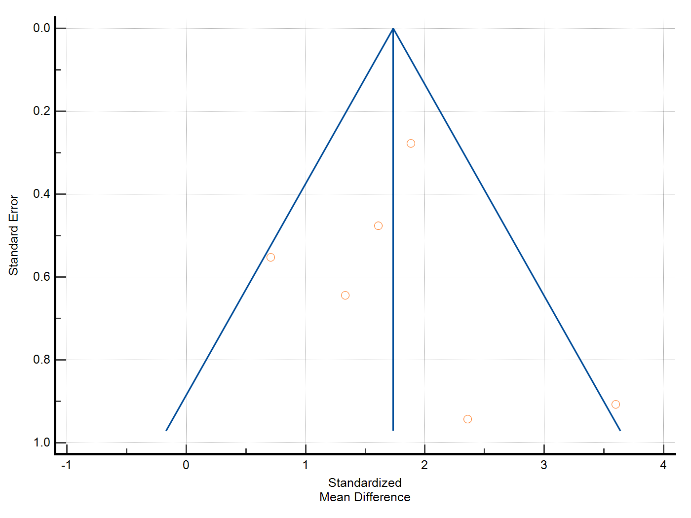


Supplementary Figure 4 - Funnel Plot of the studies showing publication bias by Egger’s test for the outcome creatinine after the treatment with PP/PE.


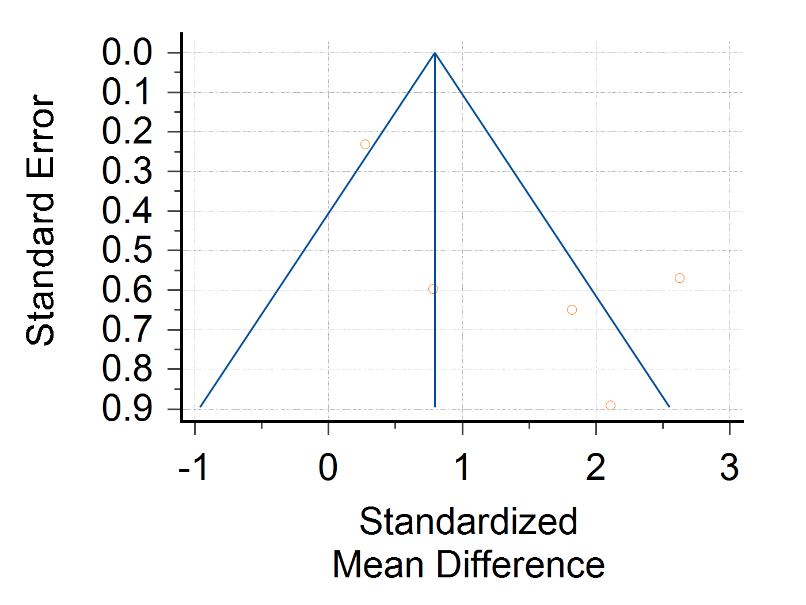


Supplementary Figure 5- Funnel Plot of the studies showing publication bias by Egger’s test for the outcome Prothrombin Time after the treatment with PP/PE.
